# Divergent Soil Aggregate Stability Despite Similar Organic Carbon Gains Under Long-Term Maize Intercropping with Different Legume Cover Crops

**DOI:** 10.3390/microorganisms14040886

**Published:** 2026-04-15

**Authors:** Tantan Zhou, Duofeng Pan, Yunpeng Zhou, Dandan Li, Jisheng Xu, Zepeng Xuan, Jiawen Deng, Jiabao Zhang, Bingzi Zhao

**Affiliations:** 1 Institute of Soil Science, Chinese Academy of Sciences, Nanjing 211135, China; 2 University of Chinese Academy of Sciences, Beijing 100049, China; 3 Institute of Forage and Grassland Sciences, Heilongjiang Academy of Agricultural Sciences, Harbin 150086, China; 4 University of Chinese Academy of Sciences, Nanjing, Nanjing 211135, China

**Keywords:** maize-legume intercropping, mean weight diameter (MWD), soil organic carbon (SOC), C proportion within macro-aggregates, soil microbial communities

## Abstract

Intercropping maize with legume cover crops has been shown to increase soil organic carbon (SOC) and alter soil microbial communities, potentially affecting soil aggregate stability. However, whether different legume cover crop varieties vary in their effects on SOC enhancement and aggregate stability improvement, and whether such variation is associated with their capacity to enhance distinct microbial taxa, remains unclear. Here, we conducted a five-year field experiment comprising maize monoculture (MM) and six intercropping systems in which maize was grown with different legume cover crop varieties. We aimed to assess the role of bacterial, non-AMF, and arbuscular mycorrhizal fungal (AMF) community composition in influencing SOC and aggregate stability, measured as mean weight diameter (MWD). On average, the six intercropping systems significantly increased SOC by 28% compared with MM, with no significant differences among legume varieties. However, MWD varied significantly depending on the specific legume used. Specifically, intercropping with red clover or sesbania resulted in MWD values similar to MM, whereas intercropping with soybean, hairy vetch, common vetch, or yellow sweet clover led to significantly higher MWD. Notably, MWD was positively correlated with the proportion of C within macroaggregates (>0.25 mm), and this effect was linked to the enrichment of specific microbial taxa—including the bacterium *RB41*, the non-AMF *Trichoderma*, and AMF (unclassified *Glomerales*, *Glomus2*, and *Glomus3*)—in systems with high MWD. These findings indicate that while SOC accrual under intercropping is robust across legume varieties, aggregate stability is contingent upon the identity of the legume and its associated microbiota. Selecting legume varieties with a greater ability to increase the abundance of specific microorganisms that enhance C allocation into macroaggregates can simultaneously improve both SOC accumulation and aggregate stability in maize-based intercropping systems.

## 1. Introduction

Cover crops refer to crops that fill the temporal or spatial gaps of bare soil during the growth period or after harvest of the main crop [1]. Extensive research has demonstrated that cover crops can enhance soil organic carbon (SOC) sequestration in main crop–cover crop systems by directly inputting organic carbon into the soil, promoting soil aggregate formation, and modulating soil microbial communities [1,2]. Among them, legume cover crops exhibit a superior effect on increasing SOC compared to non-legume cover crops [3]. Legume cover crops are often prioritized in intercropping systems with cereal grains like maize, due to their nitrogen-fixing capability, complementary resource use (e.g., C3 vs. C4 photosynthesis, spatial niche differentiation), and the favorable chemical composition (lower C: N ratio) of their residues which facilitates microbial processing [4,5]. However, it remains unclear whether different legume cover crop varieties intercropped with maize vary in their capacity to enhance SOC. Systematic evaluation through long-term in situ field trials comparing locally adapted legume cover crop varieties is therefore needed.

Soil aggregate stability refers to the ability of soil to resist external disruptive stress, directly influencing key processes such as soil water dynamics, gas exchange, nutrient cycling, and microbial activity [6,7]. Mean weight diameter (MWD) is commonly used to evaluate soil aggregate stability [8], which is derived from aggregate size distribution.

SOC has been proven as the primary factor governing aggregate stability and size distribution [9]. The C proportion in >0.25 mm macroaggregates have been identified as the most critical factor influencing aggregate stability [10]. Intercropping maize with legumes such as peanut (*Arachis hypogaea* L.) or soybean has been shown to significantly increase the C proportion in >0.25 mm macroaggregates [2,11], which is related to changes in microbial survival strategies induced by intercropping [12]. Greater AMF abundance resulting from cultivation of cover crops enhances the production of polysaccharides and glomalin, thereby improving soil aggregate stability [13]. Nevertheless, the microbial mechanisms through which intercropping maize with different varieties of legume cover crops affects aggregate stability by altering the C proportion in >0.25 mm macroaggregates remain unclear.

Soil microorganisms are key drivers of SOC turnover and aggregate formation [14]. Macroaggregates (>0.25 mm) generally harbor a higher abundance of copiotrophic bacteria, which accelerate the decomposition of exogenous organic materials and thereby promote the accumulation of C in the >0.25 mm fraction [15]. Different cover crop species can shape microbial community composition and recruit specific taxa through mechanisms such as rhizodeposition and variations in residue quality [16,17]. Xie et al. (2025) reported that the pore sheath of alfalfa was enriched with unique core taxa such as *Sphingomonadaceae*, that of rapeseed with *Saccharimonadales*, and that of a radish–hairy vetch mixture with *Chitinophagaceae* and *Verrucomicrobium*, all of which are closely associated with SOC cycling [16]. Similarly, Somenahally et al. (2018) observed that after legume cover crop cultivation, the relative abundance of *Sordariomycetes*, a dominant fungal class involved in SOC turnover, increased significantly, and AMF biomass increased by 30–70% [17]. These findings suggest that intercropping different legume cover crop varieties with maize may recruit or stimulate specific microbial taxa, which could in turn drive shifts in the C proportion in >0.25 mm macroaggregates.

The Northeast Black Soil Region of China is the country’s largest commercial grain base, supplying one-quarter of the national grain output and one-third of its grain transfers [18]. Maize accounts for more than 60% of the total grain production in this region [19]. However, intensive cultivation has led to a decline in SOC, deterioration of soil structure, and microbial community imbalance [20,21]. To enhance SOC, intercropping maize with legume cover crops has been widely adopted in this region [22,23]. Nevertheless, the similarities and differences among commonly used legume cover crop varieties in improving SOC and soil aggregate stability remain unclear. We hypothesize that the effects of different legume cover crops intercropped with maize on SOC and aggregate stability may be primarily driven by the capacity of specific legume varieties to stimulate and/or recruit C into different aggregate size fractions via specific microbial taxa. To test this hypothesis, a five-year field experiment was conducted, comparing maize monoculture with six maize-legume cover crop intercropping systems. The objectives were to (1) quantify the impacts of different maize-based intercropping systems on SOC, soil aggregate mass and aggregate-associated C distribution, and the composition of soil bacterial, non-AMF, and AMF communities, (2) elucidate the associations among soil aggregate stability, aggregate-associated C distribution, and microbial communities, and (3) identify specific microbial taxa that regulate the incorporation of C into different aggregate size classes and aggregate stability. This study provides a mechanistic understanding of how legume identity shapes soil C sequestration pathways, offering insights for optimizing cover crop selection in sustainable agroecosystems.

## 2. Materials and Methods

### 2.1. Study Site and Experimental Design

A long-term field experiment was conducted at the Heilongjiang Academy of Agricultural Sciences, Daowai District, Heilongjiang Province, China (126°51′ E, 45°50′ N). The site has a typical temperate continental monsoon climate, with annual average temperatures of 3.5 °C and an annual average precipitation of 533 mm, 80% of which falls occurs from June to September. The study area is located on the Songnen Plain in Northeast China, where soils developed on Quaternary sediments are classified as Phaeozem according to the FAO. Prior to the experiment, the topsoil (0–20 cm) had the following properties: pH 7.22, SOC 17.21 g kg^−1^, total N 1.47 g kg^−1^, total P 1.07 g kg^−1^, alkali-hydrolyzable N 151.1 mg kg^−1^, available P 51.0 mg kg^−1^, and available K 200 mg kg^−1^.

A randomized block design with seven cropping systems and three replicates (21 plots total) was established in May 2018. Each plot measured 5.2 m × 5 m and was separated from adjacent plots by 0.3 m-wide ridges on all four sides. The cropping systems are abbreviated as follows: MM, maize (*Zea mays* L.) monoculture; MI1, maize/red clover (*Trifolium pratense* L.) intercropping; MI2, maize/sesbania (*Sesbania cannabina* (Retz.) Pers.) intercropping; MI3, maize/soybean (*Glycine* max (L.) Merr.) intercropping; MI4, maize/hairy vetch (*Vicia villosa* Roth.) intercropping; MI5, maize/common vetch (*Vicia sativa* L.) intercropping; and MI6, maize/yellow sweet clover (*Melilotus officinalis* (L.) Lam.) intercropping. For maize, the inter-row distance was 0.65 m, and the inter-plant distance was 0.20 m, resulting in a planting density of 76,900 plants ha^−1^. The inter-row distance for cover crops was 0.65 m, which was planted by broadcast sowing in each row at rates of 150, 180, 150, 120, 75, and 75 kg ha^−1^ for red clover, sesbania, soybean, hairy vetch, common vetch, and yellow sweet clover, respectively. Maize monoculture plots consisted of eight rows of maize, while intercropping plots were arranged in a 4:4 strip intercropping pattern, with four rows of cover crops adjacent to four rows of maize. In the subsequent growing season, the positions of the maize strips and cover crop strips within the intercropping plots were interchanged for cultivation.

Maize and cover crops were simultaneously sown in May each year. In both monocropping and intercropping plots, the maize strips were fertilized according to local farmers’ conventional fertilization standards, with application rates of N, P_2_O_5_, and K_2_O at 150, 75, and 75 kg ha^−1^, respectively. Forty percent of the N fertilizer was applied as basal fertilizer, and the remaining 60% was top-dressed at the bell-mouthed stage. Both P and K fertilizers were applied as basal fertilizers. No chemical fertilizers were applied to the cover crop strips in any of the intercropping plots. In July each year, cover crops were harvested at their maximum biomass, chopped, and returned to the field. In October, maize was harvested for grain at maturity, with all residues chopped and returned to the field.

### 2.2. Soil Sampling and Analysis

In July 2022, prior to the cover crop harvest, intact soil cores were collected from all plots. Within each replicate plot, six soil cores (5 cm in diameter, 0–20 cm depth) were taken following an S-shaped sampling pattern. The cores were gently fragmented along natural break points and sieved (<8 mm) to remove visible plant residues and stones. The resulting soil sample was thoroughly mixed to obtain a single composite sample per plot and subsequently air-dried for aggregate fractionation. Concurrently, bulk soil samples were collected using a soil auger (5 cm diameter) at the same sampling points. For each plot, the six bulk subsamples were combined to form one composite sample, which was then sieved (<2 mm) to remove plant leaves, roots, and stones. Each composite sample was divided into two aliquots. One was air-dried for analysis of soil organic carbon (SOC), and the other was stored at −80 °C for DNA extraction.

Soil aggregate fractions were measured using the wet sieving method [24]. Briefly, 60 g of the soil samples (<8 mm) was placed on a 2 mm sieve, immersed in distilled water for 5 min, then oscillated vertically 50 times (3 cm amplitude) over 2 min using a thermostat sieve shake (DIK-2012, Daiki, Saitama, Japan). This procedure was sequentially repeated to separate the soil sample into four aggregate size classes: >2 mm macroaggregates, 0.25–2 mm macroaggregates, 0.053–0.25 mm microaggregates, and <0.053 mm silt-and-clay fractions. The four aggregate fractions were oven-dried at 60 °C to constant weight and then weighted. SOC concentration in the bulk soil and each aggregate fraction was measured by K_2_CrO_4_-H_2_SO_4_ oxidization [25]. *R*_0.25_-mass, calculated using Equation (1), represents the percentage of total soil mass contained within >0.25 mm aggregates. *R*_0.25_-SOC, computed via Equation (2), denotes the percentage of total SOC contained within >0.25 mm aggregates. Soil aggregate stability was evaluated by the mean weight diameter (MWD) determined using Equation (3).

(1) $$R_{0.25}\text{-}\mathrm{mass}=\frac{m_{i>0.25}}{m_{t}}$$

(2) $$R_{0.25}\text{-}\mathrm{SOC}=\frac{{\mathrm{SOC}}_{i>0.25}}{{\mathrm{SOC}}_{t}}$$

(3) $$MWD=\sum_{i=1}^{n}\left({\bar{x}}_{i}\right)w_{i}$$

where $m_{i>0.25}$ is the mass within >0.25 mm aggregates (g); $m_{t}$ is the total mass of the four aggregate fractions (g); ${\mathrm{SOC}}_{i>0.25}$ is the organic C retained within >0.25 mm aggregates (g); ${\mathrm{SOC}}_{t}$ is the total organic C retained within the four aggregate fractions (g); n is the number of aggregate fractions; ${\bar{x}}_{i}$ is the mean diameter of the i-th aggregate fraction (mm); $w_{i}$ is the mass proportion of the i-th aggregate fraction relative to the total mass of the four aggregate fractions.

### 2.3. Soil DNA Extraction and Amplicon Sequencing

Soil DNA was extracted from 0.5 g of soil using the Fast^®^DNA Spin Kit (MP Biomedicals, Santa Ana, CA, USA) according to the manufacturer’s instructions. DNA quality and quantity were assessed using a Nano Drop^TM^ 2000 spectrophotometer (Thermo Scientific, Waltham, MA, USA).

The bacterial 16S rRNA V4-V5 hypervariable region was amplified with primer pair 515-F (5′-GTGCCAGCMGCCGCGGTAA-3′) and 907-R (5′-CCGTCAATTCMTTTRAGTTT-3′) [26]. The fungal ITS1 region was targeted with the primers ITS1-F (5′-CTTGGTCATTTAGAGGAAGTAA-3′) and ITS2-R (5′-GCTGCGTTCTTCATCGATGC-3′) [27]. The arbuscular mycorrhizal fungi (AMF) 18S rRNA V4-V5 region was amplified with AMV4.5NF (5′-AAGCTCGTAGTTGAATTTCG-3′) and AMDGR (5′-CCCAACTATCCCTATTAATCAT-3′) [28].

PCR amplification was performed in a 25 μL reaction mixture, containing 5 μL of 5× reaction buffer, 5 μL of 5× GC buffer, 2 μL of dNTPs (2.5 mM), 1 μL of each forward and reverse primer (10 μM), 0.25 μL of DNA polymerase (5 U μL^−1^), 2 μL of genomic DNA template, and 8.75 μL of ddH_2_O. The PCR conditions were as follows: initial denaturation at 95 °C for 3 min, followed by 27 cycles consisting of denaturation at 95 °C for 30 s, annealing at 55 °C for 30 s, and extension at 72 °C for 40 s, with a final extension at 72 °C for 10 min. PCR products were verified by 2% agarose gel electrophoresis and purified using the AxyPrep DNA Gel Extraction Kit (Axygen Biosciences, Union City, CA, USA). Purified amplicons were quantified using a NanoDrop^TM^ 2000 spectrophotometer (Thermo Scientific, Waltham, MA, USA) and pooled in equimolar amounts. Sequencing libraries were subjected to paired-end sequencing on an Illumina MiSeq platform following the standard protocols of Magigen Biotechnology Co., Ltd. (Guangzhou, China). Raw sequencing data were archived in the NCBI Sequence Read Archive under accession number PRJNA1271265.

### 2.4. Bioinformatic Analysis

Raw sequencing data were processed in QIIME 2(version 2022.2) [29] using the DADA2 plugin for primer removal, denoising, quality filtering, merging, and chimera removal to identify amplicon sequence variants (ASVs) [30]. Taxonomy was assigned to ASVs using a Naive Bayes classifier trained on the SILVA database (for bacteria), UNITE database (for fungi), and MaarjAM database (for AMF). We removed ASVs classified as Glomeromycota, which belong to arbuscular mycorrhizal fungi (AMF), from the ITS1 dataset and defined the remaining sequences as non-AMF [28,31]. Samples were rarefied to an equal sequencing depth (based on the sample with the lowest sequencing depth) to minimize potential bias in downstream analyses.

### 2.5. Statistical Analysis

One-way ANOVA was conducted using SPSS Statistics 22.0 to assess significant differences in soil aggregates mass distribution, *R*_0.25_-mass, MWD, soil aggregate-associated C concentration, soil aggregate-associated C distribution, and *R*_0.25_-SOC, using Duncan’s test (*p* < 0.05). Spearman correlations examined relationships between MWD and soil aggregate-associated C distribution. Principal coordinate analysis (PCoA) based on Bray–Curtis dissimilarity was used to visualize variations in bacterial, non-AMF, and arbuscular mycorrhizal fungal (AMF) communities, while permutational multivariate analysis of variance (PERMANOVA) was performed to test for significant differences among communities, using the “vegan” package in R (version 4.2.1).

Co-occurrence networks of the bacterial, non-AMF, and AMF communities were constructed based on the dominant genera (relative abundance > 0.1%), with Spearman’s correlation coefficient calculated by the “Hmisc” R package. The Benjiamini-Hochberg approach false discovery rate was used to adjust the *p* values, and we only focused on the microbial taxa with high correlations (|r| > 0.7, *p* < 0.05). Modules in microbial co-occurrence networks are ecological clusters identified through modularity, representing functional microbial communities that occupy similar ecological niches [32]. Modularity facilitates the identification of key microbial taxa closely associated with soil nutrient cycling [33]. Modules were detected via “cluster_walktrap” in the “igraph” R package, and then major modules (within total number nodes > 10% of the total network) were identified. The network was visualized using the interactive platform Gephi (version 0.9.2) [34]. Keystone modules were identified based on Spearman analysis between C proportion in different aggregate size classes and the relative abundances of major modules (*p* < 0.05). Keystone genera were selected that genera accounted for >75% of the total abundance within each keystone module and exhibited significant differences in abundance between treatments (Duncan’s test, *p* < 0.05). Random Forest modeling was performed to identify the most crucial microbial genera for C proportion in >2 mm aggregates, C proportion in 0.25–2 mm aggregates, C proportion in 0.053–0.25 mm aggregates, and <0.053 mm silt-and-clay fractions using “randomforest” and “rfPermute” R packages. The most crucial microbial genera were visualized using the “pheatmap” R package. These genera (i.e., *RB41* [bacterium], *Trichoderma* [non-AMF], unclassified *Glomerales*, *Glomus2*, and *Glomus3* [AMF]) were subjected to principal component analysis (PCA) based on Euclidean distances. The majority of variation was represented along the first principal component (PC1), which accounted for 74.06% of the total variance. The resulting PC1 scores were then incorporated into the subsequent partial least squares path modeling (PLS-PM). Finally, PLS-PM was conducted to disentangle the direct and indirect relationships among the most crucial microbial genera (represented by PC1 scores), aggregate mass distribution, SOC concentration in each aggregate fraction, and aggregate-associated C distribution to the MWD using the “plspm” R package.

## 3. Results

### 3.1. Soil Aggregate Mass Distribution and Stability

**Figure 1:**
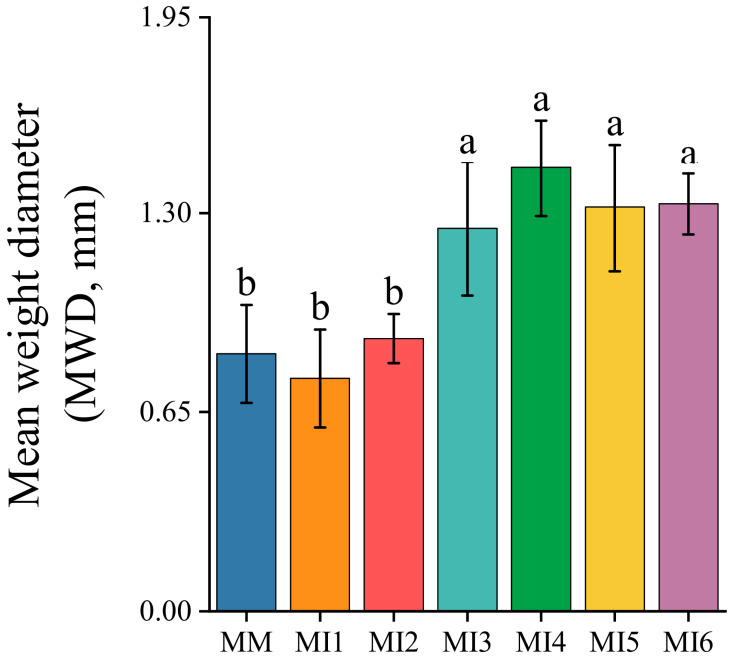
Effect of maize-based intercropping system on mean weight diameter (MWD). Error bars indicate standard deviations (*n* = 3). Different lowercase letters above bars indicate significant differences among treatments within each parameter (*p* < 0.05). The cropping systems are abbreviated as follows: MM, maize monoculture; MI1, maize/red clover intercropping; MI2, maize/sesbania intercropping; MI3, maize/soybean intercropping; MI4, maize/hairy vetch intercropping; MI5, maize/common vetch intercropping; and MI6, maize/yellow sweet clover intercropping.

Table 1 shows that after intercropping maize with six legume cover crops, the soil aggregate composition was divided into two significantly different groups. Group 1 consisted of the MI1 and MI2 treatments, while Group 2 included the MI3, MI4, MI5, and MI6 treatments. Within each group, there were no significant differences in the mass proportion of aggregates at each size class or in the *R*_0.25_-mass value. Compared with the MM treatment, the mass proportion of >2 mm aggregates in Group 1 was similar to that in MM, but significantly lower than that in Group 2 by 48% ± 5%. The average mass proportion of 0.25–2 mm aggregates from Group 1 and Group 2 treatments was significantly increased by 53% ± 24% compared with the MM treatment, with the increase in Group 1 being 9 percentage points lower than that in Group 2. For both Group 1 and Group 2 treatments, the mass proportion of 0.053–0.25 mm aggregates was significantly reduced by 43% ± 7% compared with the MM treatment, and the reduction was similar between the two groups. The mass proportion of <0.053 mm silt-and-clay fractions in Group 1 was higher than that in the MM treatment by 146% ± 42%, while no significant difference was observed between Group 2 and the MM treatment. The calculated *R*_0.25_-mass in Group 1 treatments was comparable to that in the MM treatment and 31% ± 2% lower than that in Group 2. This means that Group 1 treatments had a significantly lower mass proportion of >0.25 mm macroaggregates than Group 2, leading to reduced soil aggregate stability in Group 1 (Figure 1). As shown in Figure 1, the MWD values in Group 1 treatments did not differ significantly from those under MM treatment, but were 39% ± 7% lower than those in Group 2 treatments.

### 3.2. Soil Aggregate-Associated C Distribution

As shown in Table 2, compared with MM, intercropping maize with legume cover crops significantly increased the SOC concentration in four aggregate size fractions, with an overall increase ranging from 20% to 38%. The SOC concentrations in the 0.25–2 mm aggregates from the Group 1 treatments were higher by 11% ± 2% (*p* < 0.05) than those in Group 2 treatments. In contrast, no significant differences were observed between the two groups for the SOC concentrations in the other three aggregate fractions.

The six intercropping systems showed no significant differences in bulk soil total SOC (Table 3, *p* > 0.05). However, the average value across these systems was 28% ± 2% higher than that under the MM treatment (*p* < 0.05). The distribution of aggregate-associated C across treatments (Table 3) followed a pattern similar to that of aggregate mass distribution (Table 1). Within Group 1, the two treatments showed comparable proportions of aggregate-associated C; likewise, the four treatments in Group 2 exhibited similar proportions among themselves. However, differences in aggregate-associated C proportion between the two groups varied depending on the aggregate size class (Table 3).

Compared with the MM treatment, the proportion of C in >2 mm aggregates under Group 1 treatments did not differ significantly from MM, but was significantly lower (by 42% ± 16%) than that under Group 2 (Table 3). In the 0.25–2 mm aggregates, all treatments in both groups significantly increased the C proportion by 45% ± 18% relative to MM; however, the increase in Group 1 was 24 percentage points lower than that in Group 2. For the 0.053–0.25 mm fraction, all treatments significantly reduced the aggregate-associated C proportion by 40% ± 9% compared with MM, with no significant difference observed between the two groups. In the <0.053 mm silt-and-clay fractions, Group 1 treatments increased the C proportion by 140% ± 33% relative to MM, whereas Group 2 showed no significant difference from MM. The calculated *R*_0.25_-SOC values indicated that Group 1 treatments were similar to MM but were 24% ± 1% lower than Group 2 (*p* < 0.05).

These results demonstrated that although continuous maize intercropped with different legume cover crops over multiple years enhanced SOC to a similar extent, the distribution of SOC among aggregate size classes varied with cover crop variety. That is, under the experimental conditions of this study, the proportion of aggregate-associated C in >0.25 mm macro-aggregates was significantly lower in Group 1 treatments than in Group 2 treatments.

### 3.3. Relationship Between MWD and Soil Aggregate-Associated C Distribution

**Figure 2:**
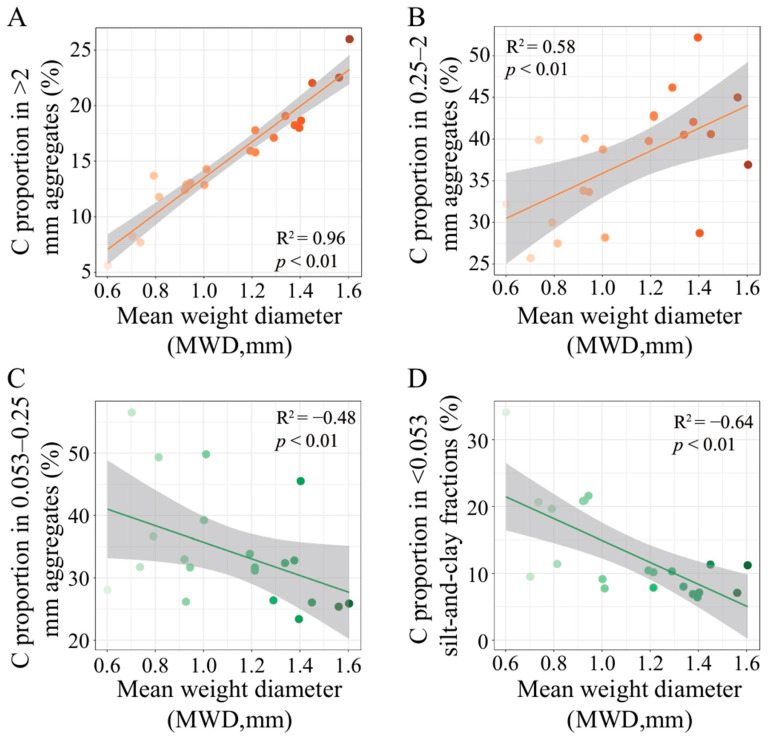
Spearman correlation analysis showing the relationships between the MWD and the C proportion in aggregate fractions of >2 mm (**A**), 0.25–2 mm (**B**), 0.053–0.25 mm (**C**), and the <0.053 mm silt-and-clay fractions (**D**). The color intensity of the dots increases with increasing MWD values, and the gray shaded areas represent the 95% confidence intervals of the fitted regression lines.

Figure 2 demonstrates a significant positive correlation between the MWD and the proportion of C in both the >2 mm and 0.25–2 mm aggregate fractions. Conversely, the MWD was significantly negatively correlated with the proportion of C in the 0.053–0.25 mm aggregates and in the <0.053 mm silt-and-clay fractions. This indicates that soil aggregate stability was strongly correlated with the sequestration of soil C by >0.25 mm macro-aggregates. Based on the results in Figure 1 and Figure 2, Table 3 the lower aggregate stability in Group 1 compared to Group 2 treatments can be attributed mainly to a smaller proportion of newly added carbon entering the >0.25 mm macro-aggregates after maize-based intercropping, despite no significant difference in SOC content was found between the two groups.

### 3.4. Soil Microbial Communities

#### 3.4.1. Composition of Soil Bacterial, Non-AMF, and AMF Communities

The dominant phyla within the bacterial community were Actinobacteriota, Proteobacteria, Acidobacteriota, Chloroflexi, and Gemmatimonadota, collectively accounting for 86.73% of the total bacterial abundance (Figure S1A). For the non-AMF community, the dominant phyla were Ascomycota, Mucoromycota, and Basidiomycota, which together constituted 87.46% of the total fungal abundance (Figure S1B). Furthermore, the AMF community was predominantly composed of Glomeraceae, Paraglomeraceae, unclassified Glomeromycota, unclassified Glomerales, and unclassified Glomeromycetes, representing 99.87% of the total AMF abundance (Figure S1C).

Principal coordinates analysis (PCoA) revealed that the bacterial, non-AMF, and AMF communities all exhibited pairwise significant differences among the seven treatments (Figure S2). These differences were further confirmed by PERMANOVA tests (Table S1).

#### 3.4.2. Soil Bacterial, Non-AMF, and AMF Co-Occurrence Networks and Ecological Clusters

**Figure 3:**
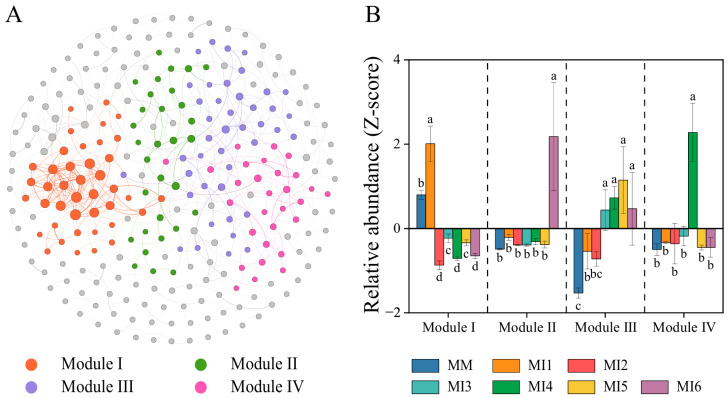
Bacterial, non-AMF, and AMF co-occurrence network based on the correlation analysis (**A**). Edges stand for strong (Spearman’s | r| > 0.7) and significant (*p* < 0.05) correlations. The network was colored by the modules, and modules I–IV were the four closely interconnected clusters. Relative abundance (z-score of accumulating abundance) of each module within the bacterial, fungal and AMF co-occurrence network (**B**) Vertical dashed lines indicate the boundaries between different modules (Modules I–IV). Bars with different lowercase letters indicate significant difference at *p* < 0.05 according to Duncan’s test. The cropping systems are abbreviated as follows: MM, maize monoculture; MI1, maize/red clover intercropping; MI2, maize/sesbania intercropping; MI3, maize/soybean intercropping; MI4, maize/hairy vetch intercropping; MI5, maize/common vetch intercropping; and MI6, maize/yellow sweet clover intercropping.

Co-occurrence networks encompassing bacterial, non-AMF, and AMF communities were constructed to identify major Modules of microbial taxa that are closely interrelated and occupy similar ecological niches (Figure 3). Four major ecological clusters (Modules I–IV) were identified within the current network (Figure 3A). Within major Module I, the relative abundances under MM and MI1 treatments were higher than those under other treatments (Figure 3B). The distribution pattern of MI6 within major Module II was similar to that of MI4 within major Module IV, i.e., their abundances were both significantly higher than those in the remaining six treatments (Figure 3B). Within major Module III, the relative abundances of all six maize-based intercropping treatments were significantly greater than that of the MM treatment. Meanwhile, within major Module III, the relative abundances in Group 2 treatments (MI3–MI6) were significantly higher than those in Group 1 treatments (MI1 and MI2) (Figure 3B, *p* < 0.05).

Table 4 indicates that C proportion in >2 mm aggregates was significantly correlated with the abundances of both major Modules I and III. Meanwhile, the C proportion in the 0.25–2 mm and 0.053–0.25 mm aggregate fractions showed significant correlations with major Modules II and III. Furthermore, in the <0.053 mm silt-and-clay fractions, the C proportion was significantly correlated with the abundance of major Module III. We defined the three major modules (Modules I, II, and III) that were significantly correlated with C proportions in different aggregate size classes as the keystone Modules, reflecting their role in driving variation in C distribution among aggregate sizes. Within each keystone Module, microbial genera that differed significantly among treatments were further identified as keystone genera, responsible for shifts in C proportion in the corresponding aggregate fractions. These keystone genera are presented in Figure S3. Specifically, the keystone genera were as follows: non-AMF (*Sordariomyceta* and *Eurotiomycetes*) and AMF (*Paraglomus1* and *Glomus1*) from keystone Module I; bacteria (*67-14*, *Solirubrobacter*, unclassified *Gemmatimonadaceae*, and *MB-A2-108*), non-AMF (*Trichoderma*, *Arthrinium*, *Chaetomium*, and *Pyrenocha*), and AMF (*Glomus2*) from keystone Module II; and bacteria (*Blastococcus* and *RB41*), along with AMF (unclassified *Glomerales*, *Glomus3*, and *Paraglomus3*) from keystone Module III (Figure S3).

**Figure 4:**
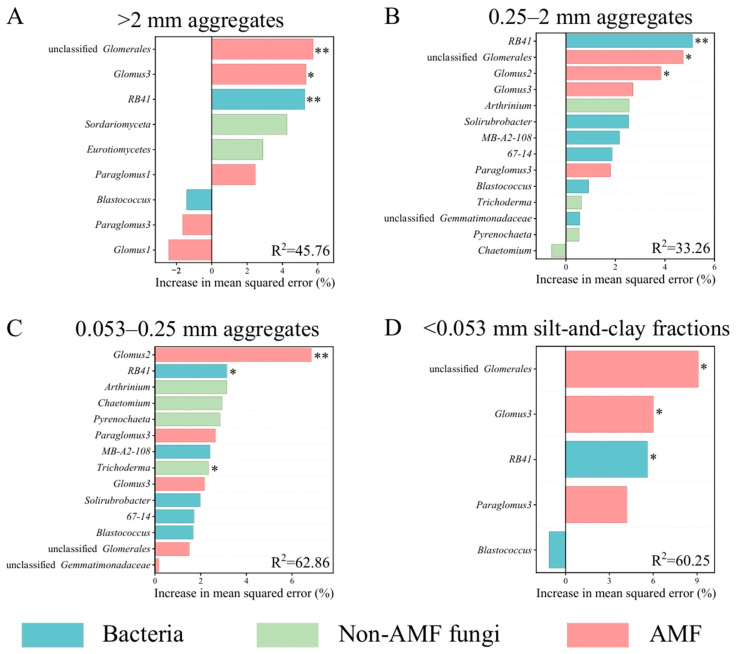
Random forest models showing the effects of keystone microbial genera on the variation in C proportion in > 2 mm aggregates (**A**), C proportion in 0.25–2 mm aggregates (**B**), C proportion in 0.053–0.25 mm aggregates (**C**), and C proportion in <0.053 mm silt-and-clay fractions (**D**) as identified by the mean squared error (MSE%) with the classification and regression tree methodology. Asterisks ** and * indicate *p* < 0.01 and *p* < 0.05, respectively.

A random forest analysis was performed to identify the most crucial microbial genera regulating C distribution across aggregate size classes. This analysis utilized data from keystone genera identified as responsible for shifts in C proportion in their respective fractions (Table 4 and Figure 4). The models accounted for 46% (C proportion in >2 mm aggregates, Figure 4A), 33% (C proportion in 0.25–2 mm aggregates, Figure 4B), 63% (C proportion in 0.053–0.25 mm aggregates, Figure 4C), and 60% (C proportion in <0.053 mm silt-and-clay fractions, Figure 4D) of the variance. Unclassified *Glomerales* and *Glomus3* (both AMF), together with the bacterium *RB41*, were the most important predictors for the variance in C proportion in >2 mm aggregates and <0.053 mm silt-and-clay fractions (Figure 4A,D). For C proportion in the 0.25–2 mm aggregates, *RB41*, unclassified *Glomerales*, and *Glomus2* were the top predictors (Figure 4B). In the 0.053–0.25 mm aggregates, *Glomus2*, *RB41*, and the non-AMF *Trichoderma* were identified as the most important explanatory factors (Figure 4C). These results indicated that the most crucial microbial genera influencing the variation in C proportion in >0.25 mm aggregates included the *RB41*, unclassified *Glomerales*, *Glomus3*, and *Glomus2*, whereas for the variation in C proportion in <0.25 mm aggregates, the key genera included *RB41*, unclassified *Glomerales*, *Glomus3*, *Glomus2*, and the non-AMF *Trichoderma*.

**Figure 5:**
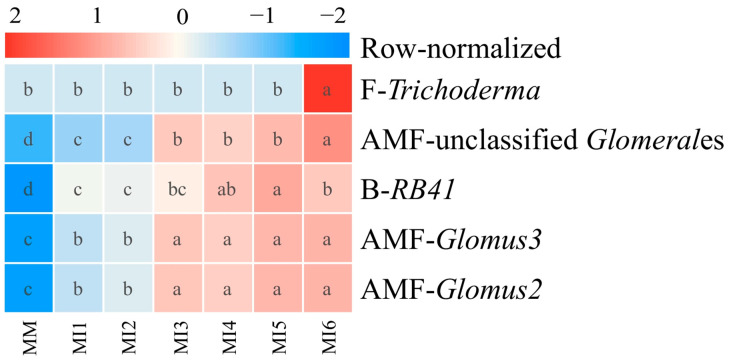
Heatmap showing the relative abundance of the five most crucial microbial genera identified by the random forest model as important regulators of C distribution across aggregate size classes. Prefixes B, F, AMF represent bacteria, non-AMF, and AMF genera, respectively. Color intensity from red to blue indicates high to low abundance. Different lowercase letters within a row denote significant differences among treatments (*p* < 0.05, Duncan’s test).

Heatmap analysis (Figure 5) revealed that, compared to the MM treatment, intercropping maize with six legume cover crops significantly increased the relative abundance of the five most crucial microbial genera regulating C distribution across aggregate size classes (Figure 4). However, the relative abundances of *RB41*, unclassified *Glomerales*, *Glomus2*, and *Glomus3* were significantly lower in Group 1 treatments than in Group 2 treatments (*p* < 0.05). In addition, *Trichoderma* abundance was significantly higher in the MI6 treatment compared to all other treatments (*p* < 0.05). These results indicated that maize intercropped with red clover or sesbania has a reduced capacity to recruit the aforementioned microbial genera relative to maize intercropped with other legume cover crops (soybean, hairy vetch, common vetch, and yellow sweet clover).

### 3.5. Linkage of MWD to the Most Crucial Microbial Genera, Aggregate Mass Distribution, SOC Concentration in Each Aggregate Fraction, and Aggregate-Associated C Distribution

**Figure 6:**
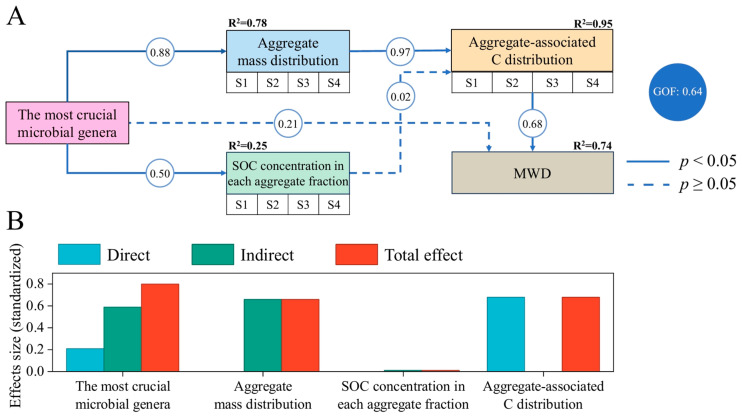
Partial least squares path modeling (PLS-PM) illustrating the direct and indirect effects of observed variables (the most crucial microbial genera) and latent variables on MWD (**A**). The latent variables include aggregate mass distribution (>2 mm aggregates [S1], 0.25–2 mm aggregates [S2], 0.053–0.25 mm aggregates [S3], and <0.053 mm silt-and-clay fractions [S4]), SOC concentration in each aggregate fraction (S1, S2, S3, and S4), and aggregate-associated C distribution (S1, S2, S3, and S4). Blue solid arrows indicate statistically significant causal relationships, whereas dashed arrows represent non-significant relationships (*p* < 0.05). Numbers on the arrows denote standardized path coefficients. R^2^ values indicate the proportion of variance in dependent variables explained by the inner model. GOF (goodness-of-fit) reflects the overall predictive performance of the model, with higher values indicating better fit. (**B**) Effects of observed and latent variables on MWD. The variation in the most crucial microbial genera was primarily represented by the first principal component (PC1), which accounted for 74.06% of the total variance.

The results of PLS-PM analysis indicated that MWD was directly and significantly positively affected by aggregate-associated C distribution (Figure 6). This distribution of aggregate-associated C was influenced by the most crucial microbial genera, primarily through their significant impact on aggregate mass distribution. Further analysis revealed that the most crucial microbial genera (total effect = 0.80) were the dominant variable affecting the change in MWD, followed by aggregate-associated C distribution (total effect = 0.68) and aggregate mass distribution (total effect = 0.66) (Figure 6B).

## 4. Discussion

### 4.1. Effects of Long-Term Maize Intercropping with Diverse Legume Cover Crops on Soil Aggregate Mass Distribution and Stability

Intercropping maize with six legume cover crops led to two distinct groups of soil aggregate mass distribution and aggregate stability, with no significant differences among treatments within each group (Table 1). The mass proportion of >0.25 mm macroaggregates and aggregate stability in Group 1 (maize/red clover and maize/sesbania) were comparable to those in maize monoculture (MM), but were significantly lower than those in Group 2 (maize/soybean, maize/hairy vetch, maize/common vetch, and maize/yellow sweet clover) (Figure 1, Table 1). The lower aggregate stability in Group 1 relative to Group 2 was primarily due to the higher mass proportions of >2 mm and 0.25–2 mm macroaggregates in Group 2 and the lower mass proportion of <0.053 mm silt-and-clay fractions in Group 1 (Figure 1, Table 1), consistent with previous findings [9,10,35].

Changes in soil aggregate mass distribution resulting from the intercropping of maize with different cover crop species may be attributed to shifts in microbial community composition, which subsequently alter the cementing agents involved in aggregate formation [28,36,37]. Maize/legume cover crop intercropping significantly increased the relative abundances of the bacterium *RB41*, the non-AMF *Trichoderma*, and the AMF unclassified *Glomerales*, *Glomus2*, *and Glomus3*. Moreover, these genera exhibited significantly higher relative abundances in Group 2 compared to Group 1 (Figure 5). *RB41* secretes substantial quantities of extracellular polymeric substances (EPS), primarily composed of carbohydrates, proteins, and DNA [38]. These EPS can function as biological cementing agents, thereby facilitating the formation of macroaggregates [37]. As a typical filamentous fungus, *Trichoderma* enhances soil particle adhesion and promotes macroaggregate formation through its extensive hyphal network and exopolysaccharide secretion [39,40]. Similarly, unclassified *Glomerales*, *Glomus2*, and *Glomus3* possess well-developed hyphal networks and contribute to macroaggregate formation via the secretion of glomalin-related soil protein [28].

Different root morphologies of cover crops result in the formation of aggregates of varying sizes through penetration and fragmentation [41,42,43]. In Group 1 treatments, red clover and sesbania are both taproot species with well-developed primary roots and strong root penetration capacity. Their root growth may disrupt soil structure and exacerbate the fragmentation of soil aggregates, thereby increasing the mass proportion of silt-and-clay fractions [42,44]. In contrast, Group 2 treatments, including soybean, hairy vetch, common vetch, and yellow sweet clover, possess well-developed fibrous root systems and fine root branches. The enhanced root entanglement in these species promotes the formation and stability of macroaggregates [43,45,46].

### 4.2. Effects of Long-Term Maize Intercropping with Diverse Legume Cover Crops on SOC and Its Distribution Across Aggregate Size Classes

Intercropping maize with six leguminous cover crops significantly and uniformly increased total SOC concentration by 28% (Table 3). This finding aligns with the results of Liu et al. (2024) and Zhang et al. (2025), who reported, based on three-year field trials, that intercropping maize with various leguminous cover crops similarly elevated SOC content by over 9%, and this increase was consistently associated with a marked enhancement in fungal necromass carbon [47,48]. The six leguminous cover crops selected in this study exhibited comparable C:N ratios (all < 20) [5], facilitating rapid microbial utilization upon incorporation. The analogous SOC increments observed across cover crop species suggest that these treatments elicited similar increases in SOC via two mechanisms: (1) stimulation of microbial growth and proliferation, leading to elevated microbial necromass carbon accumulation, and (2) enhanced decomposition and transformation of exogenous organic substrates into SOC. For instance, Zhou et al. (2025) demonstrated that, relative to straw return alone, the addition of milk vetch (*Astragalus sinicus* L.) under straw return conditions attenuated the positive priming effect while promoting the activities of r-strategists and fungi [49]. This shift facilitated the conversion of a greater proportion of straw carbon into microbial necromass carbon, thereby significantly improving carbon sequestration efficiency and increasing SOC content. Similarly, Hu et al. (2024) reported that leguminous cover cropping augmented microbial carbon transformation pathways by enhancing the availability of soil carbon and nitrogen nutrients, consequently elevating SOC content [3]. Conversely, long-term maize monoculture substantially depletes soil nutrient reserves, microbial biomass, and microbial activity. The return of maize straw with a high C:N ratio (>50) induces microbial nitrogen mining, which accelerates the mineralization of native SOC and disrupts soil structure. This process promotes the decomposition and mineralization of organic carbon, particularly diminishing organic carbon content within macroaggregates [50,51,52].

Table 3 shows that although long-term intercropping of maize with six cover crops equivalently and significantly increased SOC, the distribution of SOC across aggregate size fractions was dependent on cover crop species. Xu et al. (2026) also reported, based on a six-year field trial, that compared with maize monoculture, intercropping maize with hairy vetch or rapeseed (*Brassica napus* L.) both significantly and equally enhanced SOC content; however, the proportion of C in >0.25 mm aggregates was significantly higher under the former than under the latter [52]. Under the conditions of the present study, the patterns of aggregate-associated C distribution across aggregate size classes varied among treatments, mirroring the differences in aggregate mass distribution among treatments (Table 1 and Table 3). Specifically, the C proportion in >0.25 mm aggregates in Group 1 treatment was not significantly different from that in the maize monoculture (MM) treatment, but was significantly lower than that in Group 2 treatment. This was mainly attributed to the higher C proportions in the >2 mm and 0.25–2 mm aggregates and the lower C proportion in the <0.053 mm silt-and-clay fractions in Group 2 relative to Group 1, leading to a higher *R*_0.25_-SOC in Group 2 than in Group 1 (Table 3). Soil microorganisms are known to regulate the transformation and stabilization of exogenous organic materials into C pools across aggregate size fractions [36]. The greater enrichment of bacterium *RB41*, non-AMF fungus *Trichoderma*, and AMF unclassified *Glomerales*, *Glomus2*, and *Glomus3* in Group 2 likely contributed to the higher C proportion in >0.25 mm aggregates observed in Group 2 compared to Group 1 (Figure 4 and Figure 5). *RB41* has been demonstrated to be obligately involved in the degradation of plant-derived organic matter [53]; its abundance was significantly positively correlated with soil β-glucosidase (BG) and N-acetyl-β-glucosaminidase (NAG) activities, and it promoted SOC accumulation by enhancing C use efficiency and suppressing SOC mineralization losses [54]. *Trichoderma* can directly facilitate the decomposition of exogenous organic materials and SOC accumulation via secretion of lignocellulose-degrading extracellular enzymes [41]. Unclassified *Glomerales* and *Glomus* can recruit and activate saprophytic microorganisms through glomalin release and improvement of soil nutrient availability, thereby indirectly promoting exogenous organic matter decomposition and SOC accumulation [55,56]. Exogenous organic material-derived SOC primarily enters the >0.25 mm macroaggregates, with the C proportion in macroaggregates being significantly higher than that in the <0.25 mm aggregate fractions [24,35,57].

### 4.3. Positive Correlations Between C Proportion in Macroaggregates and MWD

MWD was significantly positively correlated with the proportion of C in >0.25 mm aggregates, while being significantly negatively correlated with the proportion of C in <0.25 mm aggregate fractions (Figure 2), indicating that an increased proportion of macroaggregate-associated C contributes to the enhancement of MWD. This finding is consistent with the results reported by Sun et al. (2023) and Zhao et al. (2023), who found that maize/peanut intercropping enhances macroaggregate formation capacity by increasing the proportion of C in >0.25 mm macroaggregates and reducing the proportion of C in silt-and-clay fractions, thereby increasing MWD [2,11].

SOC is a key stabilizing agent of aggregates [9,58]. Within macroaggregates, a substantial proportion of labile C components readily accessible to microorganisms (e.g., polysaccharides and proteins) functions as cementing agents, facilitating the aggregation and stabilization of soil particles [59]. Simultaneously, these components stimulate microbial activity, as evidenced by increased microbial biomass and hyphal proliferation, leading to the secretion of extracellular polymeric substances and adhesive materials [60]. This process further enhances the entanglement and cementation among microaggregates, thereby promoting their transformation into macroaggregates [61]. Ultimately, a dynamic feedback loop is established, driving enhanced SOC accumulation and increased MWD [10,52,62]. Additionally, macroaggregates tend to be enriched with relatively stable chemical constituents, such as methoxyl/N-alkyl C, which may further reinforce aggregate structural integrity via hydrophobic effects [63]. As the C content within macroaggregates increases, these organic materials more effectively fill and bridge soil pore spaces, reducing aggregate susceptibility to external disturbances. Consequently, overall soil structural stability is improved, resulting in elevated MWD [64].

## 5. Conclusions

Taken together, this study provides compelling evidence that intercropping maize with legume cover crops differentially influences soil aggregate stability, despite comparable enhancements in soil organic carbon (SOC) across all systems. Specifically, maize intercropped with soybean, hairy vetch, common vetch, or yellow sweet clover markedly improved aggregate stability relative to sole maize cropping, whereas maize/red clover and maize/sesbania combinations did not, exhibiting stability levels similar to the maize monoculture control. These differences were driven by a greater allocation of organic carbon into macroaggregates (>0.25 mm) in the former group, revealing a decoupling between total SOC accumulation and its distribution among aggregate fractions. Importantly, we identify specific microbial taxa associated with this preferential carbon flow, offering new mechanistic insights into the biological regulation of soil structure formation. Our findings carry significant implications for the design of targeted intercropping strategies: selecting cover crop varieties based not only on biomass production but also on their capacity to increase functionally distinct microbial communities may enhance soil physical health and carbon persistence. Furthermore, the specific microbial taxa identified here represent potential targets for microbial engineering or co-inoculation approaches to steer carbon into macroaggregates, thereby promoting soil aggregate stability. Overall, our findings underscore the importance of the capacity of legume cover crops to enhance beneficial microorganisms in mediating SOC improvement and modifying soil aggregate stability. Continuous annual monitoring is recommended in the future to further elucidate the temporal dynamics and underlying mechanisms linking soil aggregate stability and SOC content.

## Figures and Tables

**Table 1:** Soil aggregate mass distribution as influenced by maize-based intercropping systems.

	Soil Aggregate Mass Distribution (%)				
	>2 mm	0.25–2 mm	0.053–0.25 mm	<0.053 mm	*R*_0.25_-mass
MM	10.16 ± 3.00 b	22.01 ± 1.42 d	55.06 ± 3.95 a	12.77 ± 1.93 b	32.17 ± 4.16 b
MI1	7.43 ± 2.87 b	29.57 ± 3.50 c	27.71 ± 3.54 c	35.29 ± 6.51 a	37.00 ± 5.52 b
MI2	10.16 ± 1.64 b	28.38 ± 1.19 c	33.82 ± 4.46 bc	27.64 ± 2.28 a	38.54 ± 2.37 b
MI3	16.05 ± 4.80 a	34.20 ± 4.00 bc	37.73 ± 3.58 b	12.02 ± 1.48 b	50.25 ± 4.19 a
MI4	19.74 ± 3.97 a	36.85 ± 3.55 ab	27.96 ± 1.29 c	15.45 ± 0.92 b	56.59 ± 0.51 a
MI5	16.72 ± 3.51 a	38.67 ± 3.81 ab	31.64 ± 4.48 bc	12.98 ± 2.63 b	55.38 ± 7.10 a
MI6	16.12 ± 1.52 a	42.14 ± 5.42 a	30.20 ± 4.05 c	11.55 ± 2.24 b	58.25 ± 5.88 a

Data are means ± standard deviations (*n* = 3). Different lowercase letters within the same column denote significant differences (*p* < 0.05). *R*_0.25_-mass represents the percentage of total soil mass contained within >0.25 mm aggregates. The cropping systems are abbreviated as follows: MM, maize monoculture; MI1, maize/red clover intercropping; MI2, maize/sesbania intercropping; MI3, maize/soybean intercropping; MI4, maize/hairy vetch intercropping; MI5, maize/common vetch intercropping; and MI6, maize/yellow sweet clover intercropping.

**Table 2:** Effects of maize-based intercropping systems on SOC concentration within each aggregate fraction.

	SOC Concentration (g kg^−1^ Aggregate Fraction)			
	>2 mm	0.25–2 mm	0.053–0.25 mm	<0.053 mm
MM	19.63 ± 0.68 c	21.39 ± 0.34 d	16.37 ± 1.25 d	12.97 ± 1.48 c
MI1	26.22 ± 2.52 b	28.40 ± 0.81 a	23.28 ± 0.45 ab	15.88 ± 2.22 ab
MI2	30.97 ± 2.33 a	26.63 ± 1.60 a	23.31 ± 0.42 ab	17.51 ± 1.98 a
MI3	24.81 ± 2.66 b	24.14 ± 0.22 c	23.70 ± 1.74 a	15.52 ± 0.64 ab
MI4	24.65 ± 0.06 b	25.07 ± 0.51 bc	20.97 ± 0.74 c	15.90 ± 0.24 ab
MI5	24.36 ± 0.10 b	24.84 ± 0.97 bc	21.45 ± 0.31 bc	16.02 ± 0.12 ab
MI6	26.39 ± 2.81 b	25.44 ± 1.22 bc	22.67 ± 1.11 abc	14.49 ± 0.94 bc

Data are means ± standard deviations (*n* = 3). Different lowercase letters within the same column denote significant differences (*p* < 0.05). The cropping systems are abbreviated as follows: MM, maize monoculture; MI1, maize/red clover intercropping; MI2, maize/sesbania intercropping; MI3, maize/soybean intercropping; MI4, maize/hairy vetch intercropping; MI5, maize/common vetch intercropping; and MI6, maize/yellow sweet clover intercropping.

**Table 3:** Effects of maize-based intercropping systems on total SOC and its distribution across aggregate size classes.

Treatment	Total SOC (g kg^−1^)	Aggregate-Associated C Distribution (%)				
		>2 mm	0.25–2 mm	0.053–0.25 mm	<0.053 mm	*R*_0.25_-SOC
MM	16.43 ± 0.87 b	11.44 ± 0.32 cd	27.12 ± 1.27 d	51.88 ± 4.02 a	9.56 ± 1.84 b	38.56 ± 4.29 cd
MI1	21.18 ± 0.99 a	8.75 ± 3.74 d	37.38 ± 4.49 bc	28.66 ± 2.83 c	25.22 ± 7.71 a	46.13 ± 3.68 c
MI2	20.84 ± 0.19 a	13.05 ± 0.65 bc	32.49 ± 2.15 bc	33.77 ± 2.57 bc	20.69 ± 0.98 a	45.54 ± 1.61 c
MI3	20.42 ± 0.52 a	16.86 ± 3.47 abc	35.99 ± 6.37 bc	39.04 ± 6.57 b	8.10 ± 1.01 d	52.86 ± 2.21 ab
MI4	21.18 ± 0.88 a	21.71 ± 4.44 a	41.23 ± 4.66 ab	26.11 ± 0.26 c	10.95 ± 0.56 b	62.93 ± 0.34 a
MI5	21.26 ± 0.60 a	18.09 ± 3.85 ab	42.54 ± 2.63 ab	30.14 ± 4.33 c	9.24 ± 1.87 b	60.63 ± 6.15 ab
MI6	21.19 ± 0.39 a	18.01 ± 0.23 ab	45.64 ± 5.68 a	29.29 ± 5.14 c	7.06 ± 0.74 b	63.65 ± 5.67 a

Data are means ± standard deviations (*n* = 3). Different lowercase letters within the same column denote significant differences (*p* < 0.05). *R*_0.25_-SOC denotes the percentage of total SOC contained within >0.25 mm aggregates. The cropping systems are abbreviated as follows: MM, maize monoculture; MI1, maize/red clover intercropping; MI2, maize/sesbania intercropping; MI3, maize/soybean intercropping; MI4, maize/hairy vetch intercropping; MI5, maize/common vetch intercropping; and MI6, maize/yellow sweet clover intercropping.

**Table 4:** Correlations between C proportion in different aggregate size classes and the relative abundances of four major modules.

	Modul I	Module II	Module III	Module IV
C proportion in >2 mm aggregates	−0.43 *	0.26	0.76 **	0.27
C proportion in 0.25–2 mm aggregates	−0.23	0.57 **	0.60 **	0.25
C proportion in 0.053–0.25 mm aggregates	0.21	−0.59 **	−0.47 *	−0.32
C proportion in <0.053 mm silt-and-clay fractions	0.02	−0.02	−0.48 *	0.07

Asterisks ** and * indicate *p* < 0.01 and *p* < 0.05, respectively.

## Data Availability

The raw sequencing data were archived in the NCBI Sequence Read Archive under accession number PRJNA1271265. Further inquiries can be directed to the corresponding authors upon reasonable request.

## Supplementary Materials

The following supporting information can be downloaded at https://www.mdpi.com/article/10.3390/microorganisms14040886/s1. Figure S1: Relative abundance of soil bacterial phyla (A), non-AMF phyla (B), and AMF families (C). Others comprises microbial phyla/families with relative abundance below 1% and unidentified phyla/families; Figure S2: The principal coordinate analysis (PCoA) plots depicting the ASV-based Bray–Curtis distance of the soil bacterial (A), non-AMF (B), and AMF (C) communities between the treatments; Figure S3: Microbial genera exhibiting significant differences in abundance across different treatments in Module I (A), Module II (B), and Module III (C); Table S1: Permutational multivariate analysis of variance (PERMANOVA) showing pairwise differences in soil bacterial, non-AMF, and AMF community composition between different cropping systems.
