# Supplementary material for: Divergent Soil Aggregate Stability Despite Similar Organic Carbon Gains Under Long-Term Maize Intercropping with Different Legume Cover Crops
Source: Microorganisms. 2026 Apr 15;14(4):886. doi: 10.3390/microorganisms14040886 (PMC13118399; doi:10.3390/microorganisms14040886)
Supplement: Supplementary file 1 [file microorganisms-14-00886-s001.zip › microorganisms-4195322-Supplementary Materials.pdf]

Supporting Information for:

Divergent Soil Aggregate Stability Despite Similar Organic Carbon Gains under Long-Term Maize Intercropping with Different Legume Cover Crops

Tantan Zhou [1,2], Duofeng Pan [3], Yunpeng Zhou [1,2], Dandan Li [1], Jisheng Xu [1], Zepeng Xuan [1], Jiawen Deng [1,2], Jiabao Zhang [1], and Bingzi Zhao [1,4,] *

[1] Institute of Soil Science, Chinese Academy of Sciences, Nanjing 211135, China
[2] University of Chinese Academy of Sciences, Beijing 100049, China
[3] Institute of Forage and Grassland Sciences, Heilongjiang Academy of Agricultural Sciences, Harbin 150086, China
[4] University of Chinese Academy of Sciences, Nanjing, Nanjing 211135, China
* Correspondence: bzhao@issas.ac.cn

Supplementary Figure Captions

Figure S1. Relative abundance of soil bacterial phyla (A), non-AMF phyla (B), and AMF families (C). Others comprises microbial phyla/families with relative abundance below 1% and unidentified phyla/families.

Figure S2. The principal coordinate analysis (PCoA) plots depicting the ASV-based Bray–Curtis distance of the soil bacterial (A), non-AMF (B), and AMF (C) communities between the treatments.

Figure S3. Microbial genera exhibiting significant differences in abundance across different treatments in Module I (A), Module II (B), and Module III (C).

Supplementary Table Caption

Table S1. Permutational multivariate analysis of variance (PERMANOVA) showing pairwise differences in soil bacterial, non-AMF, and AMF community composition between different cropping systems.

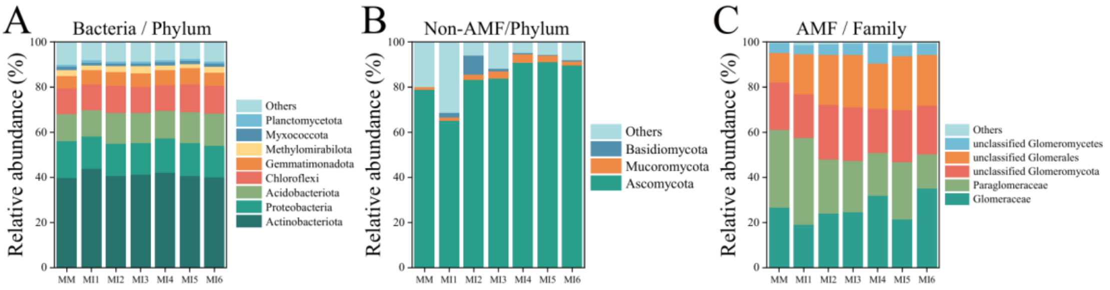

Figure S1. Relative abundance of soil bacterial phyla (A), non-AMF phyla (B), and AMF families (C). Others comprises microbial phyla/families with relative abundance below 1% and unidentified phyla/families. The cropping systems are abbreviated as follows: MM, maize monoculture; MI1, maize/red clover intercropping; MI2, maize/sesbania intercropping; MI3, maize/soybean intercropping; MI4, maize/hairy vetch intercropping; MI5, maize/common vetch intercropping; and MI6, maize/yellow sweet clover intercropping.

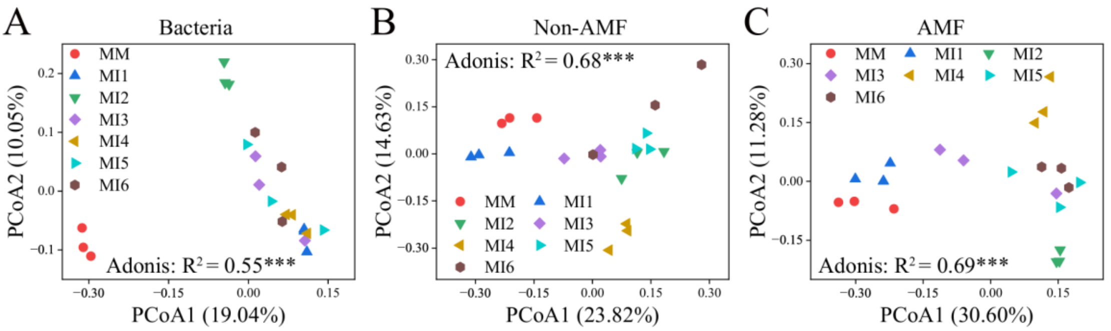

Figure S2. The principal coordinate analysis (PCoA) plots depicting the ASV-based Bray–Curtis distance of the soil bacterial (A), non-AMF (B), and AMF (C) communities between the treatments. The cropping systems are abbreviated as follows: MM, maize monoculture; MI1, maize/red clover intercropping; MI2, maize/sesbania intercropping; MI3, maize/soybean intercropping; MI4, maize/hairy vetch intercropping; MI5, maize/common vetch intercropping; and MI6, maize/yellow sweet clover intercropping.

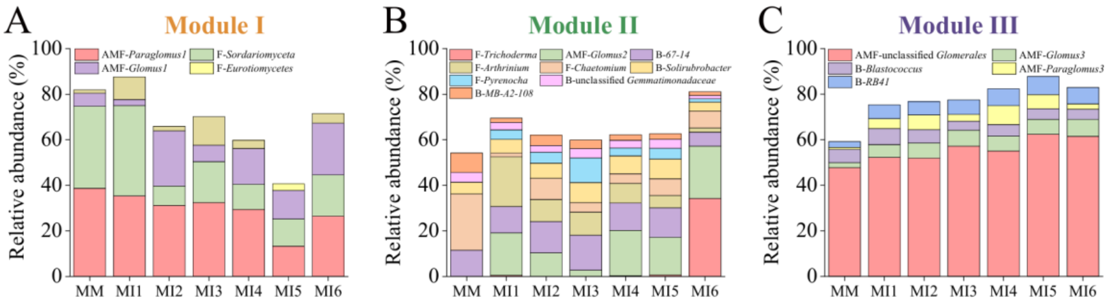

Figure S3. Microbial genera exhibiting significant differences in abundance across different treatments in Module I (A), Module II (B), and Module III (C). Prefixes B, F, AMF added before each keystone genera represent bacteria, non-AMF, and AMF respectively. The cropping systems are abbreviated as follows: MM, maize monoculture; MI1, maize/red clover intercropping; MI2, maize/sesbania intercropping; MI3, maize/soybean intercropping; MI4, maize/hairy vetch intercropping; MI5, maize/common vetch intercropping; and MI6, maize/yellow sweet clover intercropping.

Table S1. Permutational multivariate analysis of variance (PERMANOVA) showing pairwise differences in soil bacterial, non-AMF, and AMF community composition between different cropping systems.

	**Bacteria**		**Non-AMF**		**AMF**	
	R^2	*p* **value**	R^2	*p* **value**	R^2	*p* **value**
MM vs MI1	0.56	0.04	0.71	0.02	0.66	0.03
MM vs MI2	0.58	0.04	0.57	0.03	0.63	0.03
MM vs MI3	0.50	0.02	0.54	0.01	0.51	0.03
MM vs MI4	0.53	0.04	0.79	0.02	0.78	0.02
MM vs MI5	0.50	0.04	0.67	0.03	0.65	0.03
MM vs MI6	0.50	0.03	0.56	0.03	0.74	0.04
MI1 vs MI2	0.53	0.03	0.61	0.03	0.65	0.03
MI1 vs MI3	0.35	0.03	0.59	0.04	0.52	0.04
MI1 vs MI4	0.30	0.03	0.81	0.02	0.82	0.02
MI1 vs MI5	0.32	0.03	0.72	0.02	0.66	0.02
MI1 vs MI6	0.32	0.04	0.63	0.04	0.76	0.04
MI2 vs MI3	0.44	0.04	0.37	0.04	0.39	0.03
MI2 vs MI4	0.47	0.04	0.52	0.03	0.54	0.03
MI2 vs MI5	0.43	0.04	0.36	0.02	0.36	0.03
MI2 vs MI6	0.41	0.03	0.37	0.02	0.45	0.03
MI3 vs MI4	0.30	0.03	0.57	0.03	0.44	0.02
MI3 vs MI5	0.31	0.03	0.35	0.03	0.30	0.06
MI3 vs MI6	0.29	0.02	0.41	0.03	0.43	0.02
MI4 vs MI5	0.27	0.02	0.62	0.03	0.50	0.03
MI4 vs MI6	0.29	0.03	0.57	0.04	0.61	0.04
MI5 vs MI6	0.28	0.04	0.35	0.03	0.34	0.02

The cropping systems are abbreviated as follows: MM, maize monoculture; MI1, maize/red clover intercropping; MI2, maize/sesbania intercropping; MI3, maize/soybean intercropping; MI4, maize/hairy vetch intercropping; MI5, maize/common vetch intercropping; and MI6, maize/yellow sweet clover intercropping.
